# Modeling Ionic Strength Effects on Hollow-Fiber Nanofiltration Membrane Mass Transfer

**DOI:** 10.3390/membranes8030037

**Published:** 2018-07-04

**Authors:** David T. Yonge, Paul G. Biscardi, Steven J. Duranceau

**Affiliations:** 1 Jones Edmunds & Associates, 324 S. Hyde Park Ave, Suite 250, Tampa, FL 33606, USA; dyonge@jonesedmunds.com; 2 Hazen and Sawyer, 10002 Princess Palm Avenue, Suite 200, Tampa, FL 33619, USA; pbiscardi@hazenandsawyer.com; 3 Department of Civil, Environmental, and Construction Engineering, University of Central Florida, 4000 Central Florida Blvd., Orlando, FL 32816-2450, USA

**Keywords:** hollow fiber nanofiltration, ionic strength, modeling, homogeneous solution diffusion model

## Abstract

In this research, we investigated the influence of feedwater ionic strength on diffusion of divalent ions through a hollow-fiber nanofiltration membrane. The results indicated that solute flux of magnesium was increased as a result of elevating the ionic strength in the feedwater. Specifically, the feedwater ionic strength was observed to have a nonlinear impact on the diffusion of magnesium during the nanofiltration process, which was under-predicted by the homogeneous solution diffusion (HSD) model. This result suggested that elevating the feedwater ionic strength had reduced the strength of the electrostatic double layer at the membrane surface. We then developed a modification of the HSD model (referred to as the HSD-IS model) which incorporated an empirical term related to the effect of feedwater ionic strength (IS) on diffusion of magnesium. The root mean squared error of the HSD-IS model was improved by 77% as compared to the HSD model, which did not incorporate a term related to feedwater ionic strength. This improvement suggested that feedwater ionic strength should be considered when modeling hardness removal during nanofiltration.

## 1. Introduction

Nanofiltration (NF) is a pressure-driven membrane process often used in water treatment for removal of divalent metal ions. This process is often referred to as hardness removal or membrane softening. Solute mass transfer through an NF membrane is widely considered to be a diffusion-controlled process and is commonly modeled by the homogeneous solution diffusion (HSD) model which is considered the first model developed for high recovery reverse osmosis (RO) and NF systems [1]. The HSD model was developed using fundamental mass balance equations considering a single membrane element, while assuming the mass transfer of water and solutes are diffusion-based and occur due to pressure and concentration gradients, respectively. The HSD model, shown in Equation (1), incorporates the effects of feed concentration, membrane characteristics, recovery, and operational parameters to predict permeate quality [2].

(1) $$C_{p}=\frac{C_{f}K_{s}}{K_{w}\left(\Delta P-\Delta П\right)\left(\frac{2-2R}{2-R}\right)+K_{s}}$$

where,

*C_f_* = solute concentration in the feed stream (mg/L)
*C_p_* = solute concentration in the permeate stream (mg/L)
*K_w_* = mass transfer coefficient of water (gal/ft^2^-day-psi)
*K_s_* = solute mass transfer coefficient (ft/day)
*ΔP* = transmembrane pressure (psi)
ΔП = transmembrane osmotic pressure (psi)
*R* = recovery.

In this form, the HSD model can be used to predict NF process performance and permeate quality. However, the model does not account for certain physical and chemical factors and makes some simplifying assumptions common in linear and film theory modifications of the HSD model [3,4,5]. Furthermore, the HSD model assumes that the solute mass transfer coefficient is constant, despite the non-linear behavior often exhibited when varying influent water quality, operating conditions, and membrane properties [4].

Past research has proposed modifications to the HSD model to incorporate additional variables that affect mass transfer [4,6,7,8,9]. Sung [7] modified the HSD model to account for concentration polarization by incorporating the film theory model (HSDM-FT), as shown in Equation (2). This model has been used by a number of researchers, including Cussler [8] and Zhao and coworkers [4], but has not been shown to be superior to the HSD model [10,11].

(2) $$C_{p}=\frac{C_{f}K_{s}e^{F_{w}/k_{b}}}{K_{w}\left(\Delta P-\Delta П\right)\left(\frac{2-2R}{2-R}\right)+K_{s}e^{F_{w}/k_{b}}}$$

where, *K_b_* = solute mass transfer coefficient (ft/day).

Mass transfer is known to be significantly affected by additional variables, including feed ionic strength, however few researchers have investigated the direct influence ionic strength has on solute transport in membrane processes [12]. Maung and Song [13] found the removal efficiency of diffusion-controlled membranes are not only affected by pH, temperature, system hydraulics, and water quality—but also ionic strength. Specifically, ionic strength has shown to have a significant impact on mass transfer and effective pore size [14,15]. The addition of monovalent salts in feed solutions has also shown to affect the transport of divalent ions through semipermeable NF membranes, which may be explained by the reduced strength of the electrostatic double layer at the membrane surface or by the establishment of a Donnan equilibrium across the membrane [16,17]. According to Bratels and Franks [18], when a feed solution containing both cations and anions comes in contact with a negatively charged membrane, the concentration of the cations in the membrane layer is greater than their concentration in the bulk solution. Likewise, the concentration of the anions in the membrane becomes less than that of the bulk solution, which produces the electrical potential known as the Donnan potential at the boundary between the membrane and the solution. The Donnan potential attracts cations to the membrane while repelling anions away, thus increasing anion rejection [19]. In applications focused on hardness removal where nanofiltration membranes are used under the presence of monovalent ions, Nanda and researchers [20] demonstrated, with increasing ionic strength, that the tendency of magnesium ions to reach the membrane surface increases as a result of the Donnan effect, and as a result the rejection of magnesium ions decreases. In their work, Nanda and researchers [20] observed negligible change in the rejection of monovalent sodium ions. While most of these studies have focused on nanofiltration membranes used in spiral-wound elements, recent work by Zha and coworkers [21] observed a loss of salt rejection with increasing ionic strength for a hollow fiber nanofiltration membrane.

Yuan and Kilduff [22] found the transport of charged fractions of natural organic matter (NOM), while primarily influenced by diffusion, was largely affected by ionic strength [18]. Sieving coefficients for charged NOM particles were shown to increase with increasing ionic strength in ultrafiltration membranes (UF). Similar findings for divalent ion rejection were observed by Braghetta and researchers [16]. In their work, calcium rejection was shown to decrease when elevating ionic strength from 0.01 to 0.05 molar (M) using NF membranes.

In this current study, a semi-empirical modification of the HSD model has been developed to describe mass transfer of magnesium in a hollow-fiber NF membrane by considering the effect of feed ionic strength. This effect is important given the wide application of NF membranes for hardness removal in drinking water.

## 2. Materials and Methods

### 2.1. Membrane Materials

This bench-scale study was conducted using a modified polyethersulfone (PES) hollow-fiber nanofiltration (HFNF) membrane. The manufacturer indicated that the membrane was negatively charged. The membrane module was composed of 120 fibers that each had an inner diameter of the fiber lumen of 0.8 millimeters (mm). This diameter was significantly larger than that of hollow fine RO fibers, which typically have inner diameters of 0.08 mm [23]. The resin thickness was approximately 0.025 meters (m), resulting in active length 0.25 m and an active filtration area of 0.075 square meters (m^2^). The bench-scale membrane process was operated with an inside-out, cross-flow filtration configuration during each experiment. Cross-flow velocities and water flux values were operated within the membrane manufacturer’s specifications, provided in Table 1. The molecular weight cutoff (MWCO) of the membrane was 700 Daltons (Da). The expected total organic carbon (TOC) and divalent ion rejection for the membrane were approximately 95% and 45%, respectively. 

**Figure 1:**
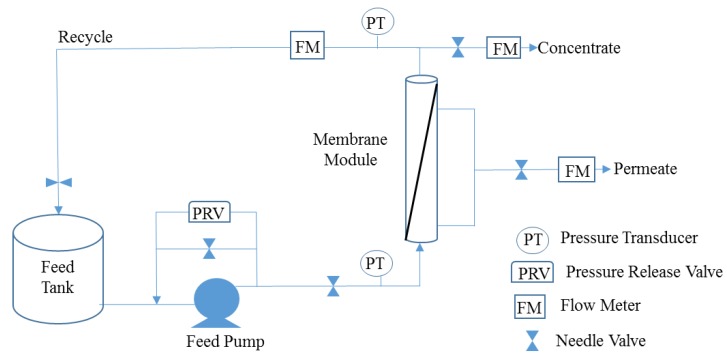
Process flow diagram of membrane testing equipment.

Each module was tested in the laboratory using the bench-scale system shown in Figure 1. This bench-scale system utilized a 5 gallon feed tank, a 0–160 pounds per square inch (psi) feed McDaniel pressure gauge (Boutte, LA, USA), a 0–30 psi permeate McDaniel pressure gauge (Boutte, LA, USA), a 0–2 gpm King flow meter (Garden Grove, CA, USA), two Omega pressure transducers (Stamford, CT, USA), one Omega thermocouple (Stamford, CT, USA), one electronic McMillan flow meter (Georgetown, TX, USA), two Optiflow 1000 Agilent Technologies bubble flowmeters (Palo Alto, CA, USA), and a Hydracell constant flow diaphragm feed pump (Minneapolis, MN, USA), with motor as shown in Figure 1.

The feed pump power requirements included 110 volts and 60 hertz. The maximum flow capacity was 1.8 gallons per minute (gpm). Ball valves with 150 psi pressure rating and ¾” schedule 80 polyvinyl chloride (PVC) piping were used under pressurized appurtenances. Plastic tubing was also used for piping where pressures were below 5 psi (such as in the permeate stream). Swagelok (Solon, OH, USA) needle valves were used for flow control. A data acquisition system was used to record feed pressure, concentrate pressure, feed temperature, and permeate flow four times per minute during experimental test runs. Recycle and concentrate flows were recorded manually. Each test run was conducted using a concentrate recycle stream with samples of the concentrate and permeate streams drawn every 5-min. Approximately 98% of each testing solution was recycled, with 2% of the solution being collected for concentrate and permeate sampling. Thus, during a given run, the feedwater would continuously concentrate, as depicted in Figure 1. 

### 2.2. Inorganic Solution Chemistry

Certified American Chemical Society (ACS) grade magnesium sulfate (MgSO_4_) and sodium chloride (NaCl) salts were used when preparing testing solutions. The first solution was composed of MgSO_4_ dissolved into deionized water to create a 5 × 10^−3^ molar (M) synthetic blend, as suggested by the membrane manufacturer. This solution was used to confirm the divalent ion removal capabilities of the hollow fiber membrane. The second and third solutions were composed of 8 × 10^−3^ M and 2 × 10^−3^ M MgSO_4_, respectively, and were used to determine if varying the concentration of the compound affected removal. These concentrations were chosen to represent waters as “very hard” and “soft water”, respectively, as described by Briggs and Ficke [24]. The fourth and fifth synthetic solutions were used to assess the effect of varying the ionic strength of the solution on membrane performance. Solutions were composed of a mixture of 5 × 10^−3^ M MgSO_4_ with NaCl concentrations of 1.25 × 10^−2^ M and 4 × 10^−2^ M, respectively. NaCl was used to elevate the ionic strength of the solutions, while maintaining a relatively constant pH at approximately 6.0. Table 2 summarizes the MgSO_4_ and NaCl concentrations, TDS, ionic strength, and hardness class of the initial solutions. The ionic strength data were calculated from the total dissolved solids of the prepared feed solutions, which were validated by measurement of the dissolved ions, conductivity, and mass balances of the permeate and concentrate streams. Duplicate experiments were conducted for each solution, which allowed for model calibration with one duplicate set and model validation with the other duplicate set. 

## 3. Results

### 3.1. Experimental Results

**Figure 2:**
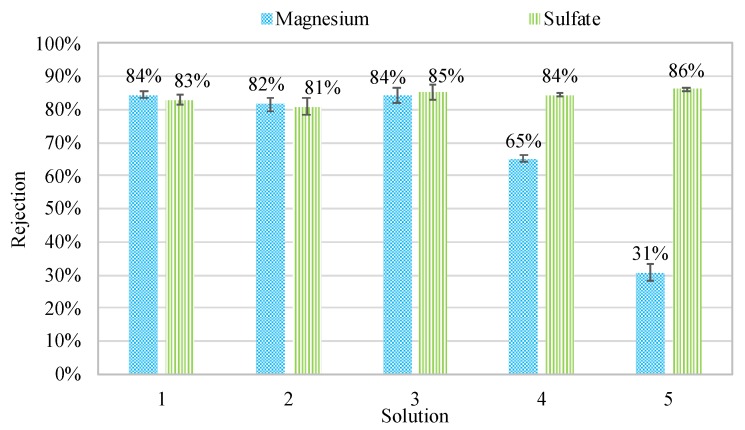
Magnesium and sulfate rejection variations for each solution.

The average rejection of magnesium and sulfate using each solution are displayed in Figure 2. Average divalent ion removal greater than 80% was achieved using solutions 1 through 3, confirming the nanofiltration properties of the hollow fiber membranes. Partial rejection of sodium and chloride was achieved (10% and 5%, respectively), however the addition of NaCl into the synthetic MgSO_4_ blends of solutions 4 and 5 caused decreased rejection with respect to magnesium. The rejection efficiency regarding magnesium decreased from 84% to 31%. On the other hand, sulfate rejection remained relatively constant, indicating that the presence of additional monovalent ions within the solutions had a specific impact on divalent cation rejection. This result suggested that the elevated ionic strength had in fact affected the charge-rejection characteristics of the membrane, in agreement with previous research [13,14,15,16,17,22].

**Figure 3:**
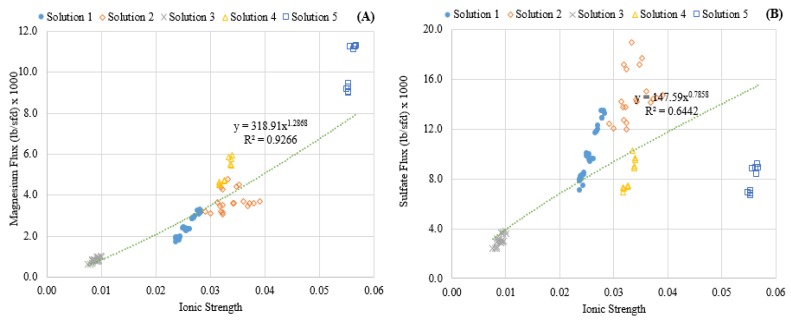
Effect of ionic strength on ion flux. (**A**) Magnesium; (**B**) Sulfate.

**Figure 4:**
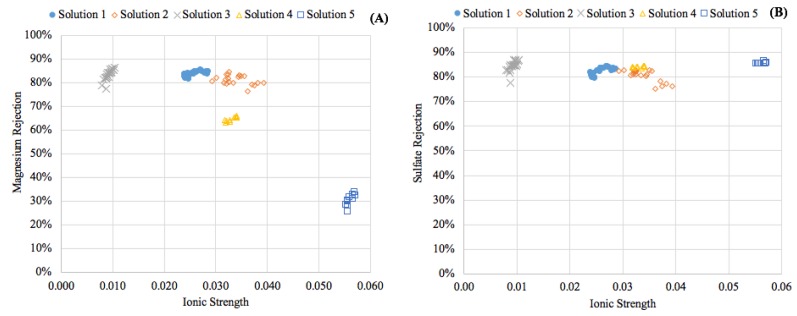
Effect of ionic strength on ion removal. (**A**) Magnesium; (**B**) Sulfate.

Figure 3 portrays the relationship between solute flux and feed ionic strength, showing increases in ionic strength had a strong non-linear correlation with magnesium passage. Conversely, sulfate flux did not appear to be correlated with ionic strength in the range investigated, as indicated by the low R-squared value. This difference suggested that the charge rejection characteristics of the membrane were affected by the higher ionic strength in the feed solution. These findings can also be observed when comparing the rejection of the solutes, as presented in Figure 4. Elevating the ionic strength significantly affected the rejection of magnesium but had little effect on the transport of sulfate across the membrane. This has been observed by Nanda and researchers [20] and was attributed to the Donnan effect. As a result, the modeling efforts presented in this work were focused specifically on the strong influence of ionic strength on magnesium diffusion.

### 3.2. Model Development

**Figure 5:**
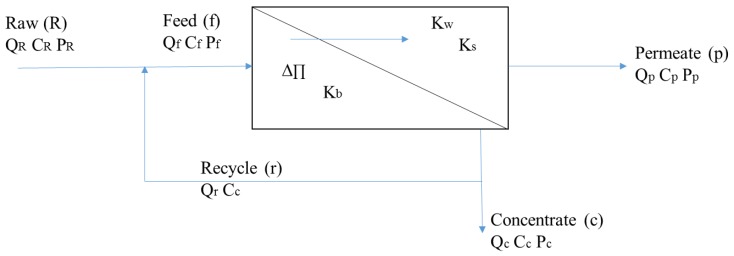
Single membrane element mass balance diagram.

To account for the effect of feed ionic strength and Donnan effect on magnesium mass transfer, a modification to the HSD model was developed. Similar to previous homogeneous solution diffusion models, the HSD-IS model considers the mathematical relationships of a single membrane element to derive an equation in terms of permeate concentration. A simplified membrane diagram has been provided in Figure 5, which illustrates the flows, solute concentrations, and pressures of the raw, feed, recycle, concentrate, and permeate streams of a single membrane element.

Assuming recycle is not implemented, Equations (3) and (4) can be derived from a mass balance around the membrane, illustrated in Figure 5. Recovery is defined as the ratio of the permeate flow to the feed flow, and is calculated using Equation (5). Typical RO processes treating seawater or hard colored ground water can achieve recoveries of 50–90%, respectively. Recoveries for microfiltration (MF) or UF systems are often greater than 90% [25].

(3) $$Q_{f}=Q_{c}+Q_{p}$$

(4) $$Q_{f}C_{f}=Q_{c}C_{c}+Q_{p}C_{p}$$

(5) $$R=\frac{Q_{p}}{Q_{f}}$$

where,

*Q* = flow (ft^3^/s)
*C* = concentration (mg/L)
Subscripts *f*, *c*, *p* = feed, concentrate, permeate.

The water flux is related to the pressure differential across the membrane by the mass transfer coefficient of water. Equation (6) describes the water flux through a membrane, accounting for the change in osmotic pressure.

(6) $$F_{w}=K_{w}\left(\Delta P-\Delta П\right)=\frac{Q_{p}}{A}$$

where,

*F_w_* = flux of water through the membrane (gal/ft^2^-day)
*K_w_* = mass transfer coefficient of water (gal/ft^2^-day-psi)
*ΔP* = transmembrane pressure (psi)
ΔП = transmembrane osmotic pressure (psi)
*A* = membrane area (ft^2^).

While solute transport in the HSD model is driven by the difference in concentration gradient between the feed and permeate sides of the membrane, the solute transport in the HSD-IS model also incorporates the feed ionic strength, as seen in Equation (7). An empirically-derived power function with two parameters (*β*1 and *β*2) was integrated into the model to account for the effect of feed ionic strength. The power function was chosen because of the non-linear effect observed in the data presented in Figure 3. This term is in addition to the solute concentration gradient term present in previously derived HSD models.

(7) $$F_{s}=K_{s}\Delta C+\beta_{1}\mu^{\beta_{2}}=\frac{Q_{p}C_{p}}{A}$$

where,

Δ*C* = [(C_f_ + C_c_)/2]—*C_p_*
*F_s_* = mass flux of solute (lb/ft^2^ · day)
*K_s_* = solute mass transfer coefficient (ft/day)
*μ* = ionic strength
*β*_1_ and *β*_2_ = constants.

The derivation of the semi-empirical HSD-IS model has been presented in steps 1 through 12 to describe solute transport with variations in ionic strength (IS) through the presence of sodium chloride. The model was developed using the HSD theory and Equations (3)–(7), which include terms for calculating water flux, solute flux, and recovery, based on the flow diagram provided in Figure 5.

1 Rearranging Equations (6) and (7) and equating yields:
  $$F_{w}C_{p}=K_{s}\Delta C+\beta_{1}\mu^{\beta_{2}}$$
2 Rearranging for Δ*C* produces:
  $$\left(\frac{C_{f}+C_{c}}{2}\right)-C_{p}=\frac{F_{w}C_{p}-\beta_{1}\mu^{\beta_{2}}}{K_{s}}$$
3 Solving for *Cc*:
  (8) $$C_{c}=\frac{2F_{w}C_{p}}{K_{s}}-\frac{2\beta_{1}\mu^{\beta_{2}}}{K_{s}}+2C_{p}-C_{f}$$
4 Rearranging Equation (3) and substituting into Equation (4) yields:
  $$Q_{f}C_{f}=Q_{p}C_{p}+\left(Q_{f}-Q_{p}\right)C_{c}$$
  Solving for *C_f_*:
  (9) $$C_{f}=\frac{Q_{p}C_{p}}{Q_{f}}+\frac{Q_{f}C_{c}}{Q_{f}}-\frac{Q_{p}C_{c}}{Q_{f}}$$
5 Substituting Equation (5) into Equation (9):
  (10) $$C_{f}=RC_{p}++C_{c}-RC_{c}$$
6 Substituting *C_c_* in Equation (10) with Equation (8) and simplifying:
  $$C_{f}=RC_{p}+\frac{2F_{w}C_{p}}{K_{s}}-\frac{2\beta_{1}\mu^{\beta_{2}}}{K_{s}}+2C_{p}-C_{f}-\frac{2RF_{w}C_{p}}{K_{s}}+\frac{2R\beta_{1}\mu^{\beta_{2}}}{K_{s}}-2RC_{p}+RC_{f}$$
7 Rearranging to group common factors *C_p_* and *C_f_* yields:
  $$C_{f}\left(2-R\right)=C_{p}\left(R+\frac{2F_{w}}{K_{s}}+2-\frac{2RF_{w}}{K_{s}}-2R\right)+\frac{2\beta_{1}\mu^{\beta_{2}}}{K_{s}}\left(R-1\right)$$
8 Solving for *C_p_*:
  $$C_{p}=\frac{C_{f}\left(2-R\right)-\frac{2\beta_{1}\mu^{\beta_{2}}}{K_{s}}\left(R-1\right)}{2-R+\frac{2F_{w}}{K_{s}}-\frac{2RF_{w}}{K_{s}}}$$
9 Factoring out $\frac{F_{w}}{K_{s}}$ and rearranging:
  $$C_{p}=\frac{C_{f}\left(2-R\right)-\frac{2\beta_{1}\mu^{\beta_{2}}}{K_{s}}\left(R-1\right)}{\frac{F_{w}}{K_{s}}\left(2-2R\right)+2-R}$$
10 Multiplying both sides by $\frac{K_{s}}{K_{s}}$ yields:
  $$C_{p}=\frac{K_{s}C_{f}\left(2-R\right)-2\beta_{1}\mu^{\beta_{2}}\left(R-1\right)}{F_{w}\left(2-2R\right)+\left(2-R\right)K_{s}}$$
11 Multiply both sides by $\frac{2-R}{2-R}$ yields:
  (11) $$C_{p}=\frac{K_{s}C_{f}-\frac{2\beta_{1}\mu^{\beta_{2}}\left(R-1\right)}{2-R}}{F_{w}\left(\frac{2-2R}{2-R}\right)+K_{s}}$$
12 Substituting Equation (6) into Equation (11) produced the HSD-IS model provided in Equation (12):
  (12) $$C_{p}=\frac{K_{s}C_{f}-\frac{2\beta_{1}\mu^{\beta_{2}}\left(R-1\right)}{2-R}}{K_{w}\left(\Delta P-\Delta \pi \right)\left(\frac{2-2R}{2-R}\right)+K_{s}}.$$

### 3.3. Numerical Simulations

**Figure 6:**
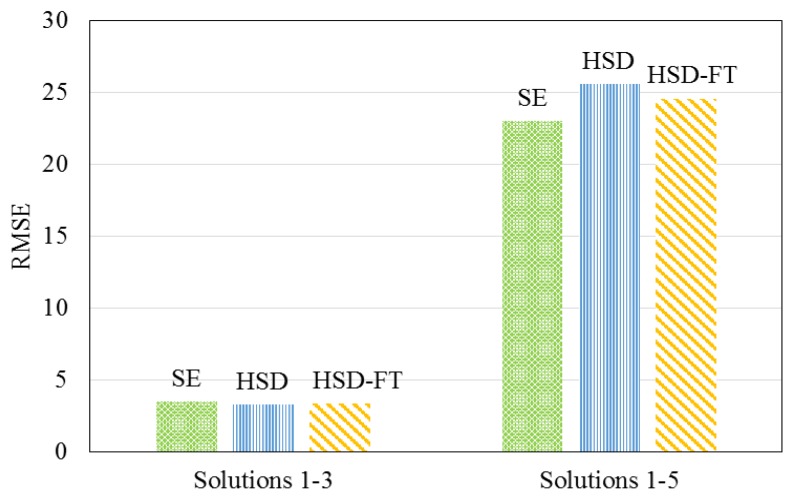
Comparison of models for describing permeate magnesium concentration.

Traditional size- and diffusion-based models were investigated in this research, including the size exclusion (SE) [26,27], HSD, and HSD-FT models to describe the data collected when testing solutions 1–5. Figure 6 displays the root mean square error (RMSE) for each model, describing the permeate magnesium concentration using experimental data from solutions 1–3 and solutions 1–5. The RMSE and residual sum of squares (SS) for each of the existing descriptive models were shown to increase, indicating that the addition of solutions 4 and 5 caused the existing models to become less accurate.

**Figure 7:**
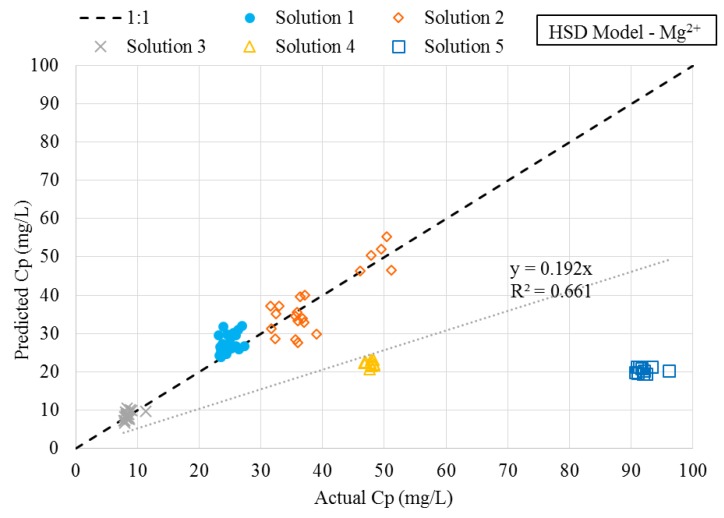
Predicted versus actual magnesium permeate concentrations using homogeneous solution diffusion (HSD) model.

**Figure 8:**
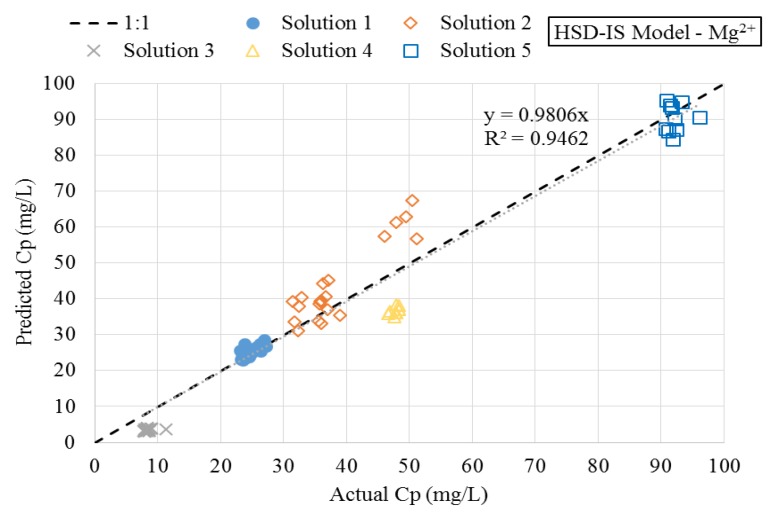
Predicted versus actual magnesium permeate concentration using HSD-IS model.

A plot of predicted versus actual magnesium concentrations using the HSD model for each solution has been provided in Figure 7. The plot confirms the data collected using solutions 4 and 5 were not described well using the HSD model. Similar results were found with the SE and HSD-FT models for magnesium, as well as each of the descriptive models for sulfate. Figure 7 depicts the predicted versus actual magnesium concentrations in the permeate stream using the newly proposed HSD-IS model. By comparison of Figure 8, significant improvement was observed when incorporating the effect of ionic strength using the HSD-IS model. 

**Figure 9:**
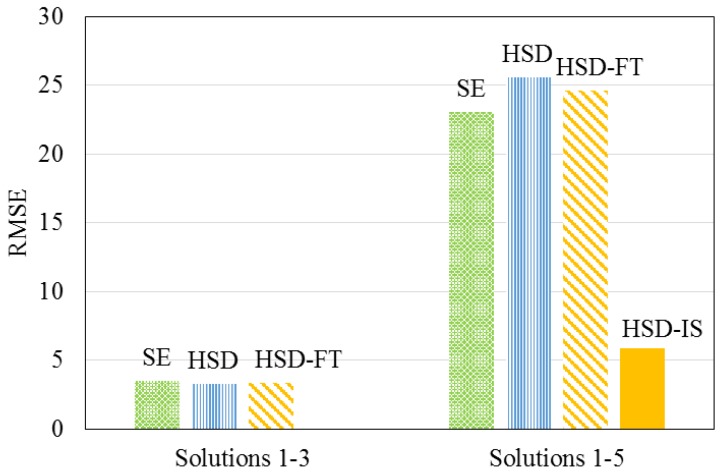
Comparison of models for describing permeate magnesium concentration.

The semi-empirical HSD-IS model was used to describe the experimental data, including solutions 4 and 5 with varying concentrations of NaCl. Model parameters were estimated by fitting experimental data to Equation (12) using least squares non-linear regression. The *β*1 and *β*2 values for magnesium were determined to be 1,044,640 and 1.83, respectively. Statistical results for the regression using the newly proposed model for predicting permeate magnesium concentration yielded a RMSE and RSS of 6 and 3129, respectively. Incorporating ionic strength into the HSD model decreased the RMSE of the predictive models by 77%, as displayed in Figure 9. While the mechanisms affecting solute transport are not fully understood, the incorporation of feed ionic strength to the HSD model improved overall model prediction using the hollow fiber NF membrane, when compared to previous models. 

## 4. Conclusions

In this study, the effect of feedwater ionic strength on divalent ion mass transport in a nanofiltration process was investigated.

- Feedwater ionic strength was observed to have a nonlinear impact on the diffusion of magnesium during a NF process.
- A modification of the HSD model was developed and proposed, which incorporated an empirical term related to the effect of feedwater ionic strength on diffusion of magnesium. This model was referred to as the HSD-IS model.
- The RMSE of the HSD-IS model was improved by 75%, as compared to existing models that do not incorporate a term related to feedwater ionic strength. This improvement, in turn, suggested that feedwater ionic strength should be considered when modeling hardness removal during nanofiltration.

Future research should investigate the applicability of this modified HSD model on reverse osmosis and spiral-wound membrane elements and consider the effects of other water quality conditions, such as varying pH, temperature, and other solutes. 

## Figures and Tables

**Table 1:** Membrane characteristics and specifications.

Parameter	Value
Operating Flux (lmh)	10–25
Cross Flow Velocity (m/s)	0.2–2.0
MWCO (Da)	700
TOC Rejection %	93–97
Divalent Ion Rejection %	30–60

**Table 2:** Initial Feed Solution Information.

Solution	MgSO_4_•7H_2_O Concentration mg/L (mM)	NaCl Concentration mg/L (mM)	TDS (mg/L)	Ionic Strength (IS)	Hardness Class
Solution 1	600 (5)	<1 (<1)	700	0.023	Hard
Solution 2	960 (8)	<1 (<1)	960	0.032	Very Hard
Solution 3	240 (2)	<1 (<1)	260	0.009	Soft
Solution 4	600 (5)	730 (12.5)	1200	0.030	Hard
Solution 5	600 (5)	2340 (40)	2600	0.054	Hard
